# Collaborative Resource Management for Negotiable Multi-Operator Small Cell Networks

**DOI:** 10.3390/s19163550

**Published:** 2019-08-14

**Authors:** Shashi Shah, Somsak Kittipiyakul, Yuto Lim, Yasuo Tan

**Affiliations:** 1 School of Information, Computer, and Communication Technology, Sirindhorn International Institute of Technology (SIIT), Thammasat University (TU), Pathum Thani 12121, Thailand; 2 School of Information Science, Japan Advanced Institute of Science and Technology (JAIST), Ishikawa 923-1292, Japan

**Keywords:** bilateral negotiation, collaboration, multi-operator, resource management, small cells

## Abstract

The ubiquitous coverage/connectivity requirement of wireless cellular networks has shifted mobile network operators’ (MNOs) interest toward dense deployment of small cells with coverage areas that are much smaller as compared to macrocell base stations (MBSs). Multi-operator small cells could provide virtualization of network resources (infrastructure and spectrum) and enable its efficient utilization, i.e., uninterrupted coverage and connectivity to subscribers, and an opportunity to avoid under-utilization of the network resources. However, a MNO with exclusive ownership to network resources would have little incentive to utilize its precious resources to serve users of other MNOs, since MNOs differentiate among others based on their ownership of the licensed spectrum. Thus, considering network resources scarcity and under-utilization, this paper proposes a mechanism for multi-operator small cells collaboration through negotiation that establishes a mutual agreement acceptable to all involved parties, i.e., a *win–win* situation for the collaborating MNOs. It enables subscribers of a MNO to utilize other MNOs’ network resources, and allows MNOs to offer small cells “as a service” to users with ubiquitous access to wireless coverage/connectivity, maximize the use of an existing network resources by serving additional users from a market share, and enhance per-user data rate. We validated and evaluated the proposed mechanism through simulations considering various performance metrics.

## 1. Introduction

The evolution in wireless cellular networks is expected to provide ubiquitous network access to users anytime and anywhere [1,2]. With the exponential growth of mobile users, the demands to provide seamless connectivity with a desired level of services to subscribers become a critical challenge for MNOs. The current small cell networks (SCNs) deployment scenarios (for both indoor and outdoor locations) usually support users of only one MNO [3], which means that, with multiple MNOs in one location, users are isolated to the separate cells of their respective MNO. To consider provision of its network resources to competitors’ users, a MNO must also consider technical aspects as well as regulatory aspects. For instance, a MNO with exclusive rights to the spectrum would have little incentive to share their precious spectrum with other MNOs as, despite significant research and regulatory efforts [4,5,6], the mobile market is mainly characterized by high upfront cost of acquiring spectrum licenses [7].

Generally, subscribers have contracts with MNOs, where they often choose the services. Service level agreement is the contract that is agreed upon in written form that generally remains static during a certain period of time. However, a MNO might not always have enough resources and/or coverage (radio networks availability), while some other MNOs might undergo under-utilization of their network resources that would often result in decreased revenue. Small cells’ users with multi-operator support could access wireless connectivity anywhere, regardless of their MNO’s network, and this in turn would enable a bring-your-own-device (BYOD) environment with uninterrupted services [3,8]. Considering network resources scarcity and under-utilization, collaborative resource management could be beneficial for MNOs by providing network access to users regardless of their coverage through BYOD environment and generating revenue from an increased market share.

In this paper, we propose a mechanism for multi-operator small cells collaboration to sustain SCN access to users in a dynamic environment, considering active users’ density and network resources availability. This would maintain desired level of services to users and avoid under-utilization of network resources. However, mutual agreement needs to be reached before any form of collaboration between self-interested MNOs (competitors) could take place. For this purpose, we consider negotiation, among potential collaborating MNOs, in order to reach to an agreement that is acceptable by all involved parties. The paper is an extension of our work in [9] with detailed and extended analysis of the multi-operator small cells collaboration mechanism, and extensive performance evaluations. The main contributions of this paper are as follows:

- The proposed mechanism of multi-operator small cells collaboration can be applicable to SCNs as well as other radio network technologies, which could offer advantages to MNOs such as: maintaining services to users even in an overloaded environment, sustaining users’ loyalty, avoiding under-utilization of available network resources, improving revenues by serving extra users from a market share, and serving users at locations not under network coverage through BYOD environment.
- Multi-operator small cells collaboration allows MNOs to offer small cells “as a service” to users, with access to wireless coverage/connectivity anywhere regardless of their operator’s network. MNOs determine their policy profile as seekers or feeders depending on the network resources availability and their utilization. Once the policy profile of the MNO is determined, negotiation trades can be established among MNOs, a seeker and a feeder, such that both the collaborating MNOs benefit upon agreement of an offer, i.e., a *win–win* situation for both participants.
- The introduced mechanism of collaboration formation, by first determining policy profile and then involving in negotiation, could also be applicable to other general fields such that rational agents would be motivated to allocate their exclusively owned resources to others.

The rest of this paper is organized as follows. Some of the related works are briefly discussed in Section 2. The mechanism for collaboration formation among multiple MNOs is introduced and discussed in Section 3. Section 4 presents the simulation results for different scenarios to demonstrate the performance gains of collaborating MNOs. Finally, Section 5 summarizes this paper.

## 2. Related Works

In the near future, the number of wireless devices accessing wireless cellular networks is expected to outnumber the human population [10]. As such, management and proper utilization of the licensed spectrum by MNOs to provide desired level of services to their subscribers will be a crucial issue [11]. In [3,12], the studies suggest that network sharing should not be taken as a threat to hinder competition among MNOs, but rather as a key approach to enhance affordable advanced services with proper regulatory framework. Initially, network sharing was exclusively viewed as an alternative to capital expenditure reduction. However, it has many other potential strategic benefits by appropriately understanding and regulating various market players [6]. Network sharing could accelerate network roll-outs for MNOs and offer enhanced services to subscribers with reduced costs. For example, in urban areas, MNOs could avoid regulatory processes for site acquisition where dense deployment restricts available space, while in rural areas, MNOs could reduce the network investment payback period.

Two network sharing standards are identified by third generation partnership project (3GPP): *multi-operator core network* (MOCN) and *gateway core network* (GWCN). In MOCN, only the radio access network (RAN) is shared where the shared RAN is directly connected to each of the MNOs’ core networks, while, in GWCN, elements of the core network are shared as well to form a shared core network so that the interconnection of the MNOs’ core networks is done at the core network level [4,5,8]. Although there are several approaches to network sharing available for MNOs, they can be simplified to *passive* and *active* sharing [13]. Passive sharing involves MNOs sharing base station (BS) sites, building premises, and masts, whereas active sharing provides virtualization of the shared physical resources: *infrastructure* and *spectrum*.

Multi-operator infrastructure sharing [13,14,15,16,17] allows the subscribers of a MNO to utilize the BS infrastructure of other MNOs, and thus the MNO makes use of another network at a place where it has no coverage or infrastructure of its own. It assumes subscribers of a MNO to freely switch between the network of multiple MNOs, and the results are shown to significantly reduce the number of BSs required to provide mobile service, and improve aggregate data rate and coverage [13,14,15]. In addition, infrastructure sharing could provide MNOs with additional benefits, such as operational cost reduction and energy saving, by considering switching-off of multiple co-located BSs [16,17]. However, an efficient and feasible mechanism needs to be designed such that, in the presence of BS infrastructure from a MNO, an entrant MNO could request on-demand utilization of the network. This could facilitate MNOs to share their infrastructures in order to maximize the use of their existing network resources while simultaneously minimizing the cost and resources requirement.

Multi-operator spectrum sharing, which refers to MNOs’ ability to share and access exclusively owned licensed spectrums of other MNOs, has been gaining significant research interests [13,15,17,18,19,20]. It provides an opportunity to maximize utilization of licensed (dedicated) spectrum that could remain under-utilized at certain time intervals. The usage of the shared spectrum among MNOs can be divided into two categories [19]: *orthogonal* and *non-orthogonal*. In orthogonal spectrum sharing, MNOs utilize a dedicated portion of spectrum from the common spectrum pool, whereas, in non-orthogonal spectrum sharing, more than one MNO simultaneously and opportunistically utilize spectrum from the common spectrum pool at any particular time. However, these studies exclusively assume prior mutual agreement among MNOs for spectrum sharing and focus only on issues such as performance comparisons [13,15,19] and radio resource allocation strategies [17,18,20].

In recent years, the principles of network sharing have evolved to a more flexible on-demand multi-tenant network [12,21,22,23,24,25,26,27,28,29], which enables MNOs to request and lease network resources from other providers dynamically via signaling. The works in [12,21,22,23,24] provide conceptual approaches that focus on design and development of general architectures for 5G platform based on concept of small cells to enable multi-tenancy coupled with an edge-based virtualized execution environment. The 3GPP-based RAN sharing concept, MOCN [4,5], can be considered as an exclusive enabler for such multi-tenancy features. The work in [25] introduces conceptual analysis and design of a signaling-based, i.e., with no human intervention, on-demand multi-tenant network architecture building on the top of the 3GPP network sharing management architecture. In their multi-tenant network architecture, a logically centralized monitoring and control entity, termed as 5G network slice broker, provides admission control for incoming requests and resource assignment by means of an enhancement of the 3GPP network sharing management architecture interfaces and service capability exposure function [30]. Conceptual architecture for multi-tenant network resource sharing and ways to coordinate the usage of resources by several entities are further discussed in [26], whereas Gang and Friderikos [27] proposed interference-based resource allocation scheme to increase utilization of the network resources among tenants. The work in [28] presents big-data-based prediction strategies for admission policy to increase profit from the network resources utilization for MNOs in a multi-tenant networks. Sciancalepore et al. [29] applied concept of stochastic geometry theory to design admission policy, i.e., radio resource allocation strategy, to the users of the tenant MNOs such that an overall throughput of the multi-tenant networks is maximized. Although the research works on multi-tenant network introduces new business models in wireless cellular networks such as allowing MNOs, with an abundance of network resources, to lease their infrastructure and/or spectrum to tenant MNOs and generate revenues from the increased market share. It is crucial to study the feasibility of multi-tenant network to leverage new business opportunities, which depend on formulating prior mutual agreements among MNOs over the usage policy of the network resources.

In general, network sharing has evolved to mobile network multi-tenancy as a novel concept that concentrates on capacity enhancement, increasing return of investment, and coverage expansion. However, MNOs’ decision on whether to enable multi-operator support relies on whether it is beneficial for their business perspective and on the risk of reducing their competitive advantage. Coverage is considered as one of the significant service attributes to subscribers and an important dimension for a MNOs to compete. Other attributes such as brand name and service innovations become an irrelevant differentiator if the MNO cannot provide coverage in the areas of subscribers’ interest. Traditionally, with macrocell coverage, infrastructures were precious and sharing the physical location with competitors could result in losing differentiation for a MNO. However, for SCNs, differentiation on such basis is less important since the deployment costs of small cells are largely reduced [31]. However, a MNO can remain a major player based on its ownership of the licensed spectrum. Thus, it is essential to formulate a mechanism where MNOs could interact and establish service level agreement over network resources usage. This paper introduces a novel mechanism of multi-operator small cells collaboration to offer an on-demand multi-operator small cells support, where a MNO negotiates with other MNO(s) over the usage of network resources and formulates mutual agreement that would provide both involved parties with some collaborative gains.

## 3. Multi-Operator SCNs: Collaboration Management Module

We consider a general network architecture of multi-operator SCNs with radio and core network elements [21,22,23] to enable multi-tenancy. The core network of a MNO is interconnected with other MNOs at core network level while the radio elements of a MNO are interconnected at a centralized location via cloud-radio access network (C-RAN). Here, the C-RAN is a logically centralized monitoring and control entity that provides a centralized network view of the underlying radio networks of a MNO. The infrastructure deployed by a MNO consists of a number of mobile stations (MSs), small-cell base stations (SBSs), C-RANs, and corresponding core networks elements.

To develop network-wide coordination and extension of services to users of multiple MNOs, the multi-operator SCNs achieve interconnection among multiple MNOs’ networks and centralized control for network-wide resource coordination via C-RANs. In C-RAN, we introduce a logical module, termed as collaboration management module (CMM), responsible for evaluating network resources and executing negotiation for collaborative formation among MNOs over on-demand network resource requests. In reference to such architecture, the CMM acts as a mediator for collaboration formation among MNOs to facilitate on-demand network resource requests and resource assignments such that the SCNs evolve to be able to provide multi-operator support to small cells’ users.

The CMM located at C-RANs in the radio network of MNOs is responsible for addressing on-demand network resource requests and assignments by establishing collaboration among multiple MNOs. In general, CMM performs two main functionalities: (i) determine a policy profile of a MNO that depends on its network resources availability and utilization; and (ii) be involved in negotiation and establish mutual agreement with other MNO(s) to utilize/provide network resources from/to others.

The provision of collaboration would ensure a MNO maintains the desired level of service to their users during changes in network resource availability, e.g., in an overloaded environment when the user’s service level may not be met, or when the MNO’s services are not available at a particular location. The multi-operator collaboration is also envisioned to create a BYOD environment that can enable services to users regardless of their parent MNO’s service/coverage and maintain ubiquitous mobile service coverage.

Considering the network resources available and their utilization, a MNO is determined to be either a *seeker* (a recipient of service access for some of its users from network resources of other MNOs), to improve services to its users, or a *feeder* (a provider of service access to some users of the seeker MNO), to improve revenue by avoiding under-utilization of its network resources. Here, we assume that revenue incurred by a small cell is calculated according to a fixed data service pricing model [32] defined in terms of average data rate perceived by each user. Specifically, the total revenue, $R_{i}$, of MNO *i* is given by,

(1) $$R_{i}=M_{i}\phi_{i}\rho_{i}^{\left(M_{i}\right)}$$

where

- $M_{i}$ is the number of users getting served by MNO *i*;
- $\phi_{i}$ is the price per-user per-unit (bps) of data rate service; and
- $\rho_{i}^{\left(M_{i}\right)}$ is the average per-user data rate offered by MNO *i* for serving $M_{i}$ users.

### 3.1. MNO’s Policy Profile

We assume that the quality of service that each MNO must strictly provide is the minimum average per-user data rate. To meet this minimum data rate, a MNO may need to use the bandwidth of other MNOs to increase the average per-user data rate above this threshold. The MNO who seeks more bandwidth is called a seeker, while those providing additional bandwidth to the seeker are called feeders.

Specifically, suppose MNO *S* serves $M_{s}$ users but the current average per-user data rate, $\rho_{s}^{\left(M_{s}\right)}$, is less than the required minimum rate, $\rho_{s,min}$. Hence, MNO *S* is a seeker since $\rho_{s}^{\left(M_{s}\right)}<\rho_{s,min}$, “condition to be a seeker”. It will need to find MNOs that can be feeders to serve some $m_{s}$ number of its current users and improve its average per-user data rate, such that it can now meet the minimum rate, i.e., $\rho_{s}^{\left(M_{s}-m_{s}\right)}\geq \rho_{s,min}$.

Suppose for simplicity only one MNO *F* is a feeder. Let $\phi_{s}^{\prime }$ be the maximum price per-user per-bps that *S* is willing to pay to the feeder. This price $\phi_{s}^{\prime }$ is called the seeker’s *reservation price*. The average per-user data rate that the feeder *F* can serve with additional $m_{s}$ users from *S* is denoted as $\rho_{f}^{\left(M_{f}+m_{s}\right)}$. Hence, *S* is willing to pay *F* at most $m_{s}\phi_{s}^{\prime }\rho_{f}^{\left(M_{f}+m_{s}\right)}$ for the service to $m_{s}$ users.

Note that after transferring $m_{s}$ users to be served by the feeder, the seeker can now serve the remaining users comfortably. That is, $m_{s}$ must be such that

(2) $$\begin{array}{c} \rho_{s}^{\left(M_{s}-m_{s}\right)}\geq \rho_{s,min} \end{array}$$

Here, by having the feeder serve $m_{s}$ users of the seeker, the seeker tries to meet its minimum rate requirement as well as improve (or not decrease) its revenue. That is, the new revenue to the seeker, with *F* serving its additional $m_{s}$ users, must not be less than the current revenue. Thus,

(3) $$\begin{array}{c} \left(M_{s}-m_{s}\right)\phi_{s}\rho_{s}^{\left(M_{s}-m_{s}\right)}+m_{s}\phi_{s}\rho_{f}^{\left(M_{f}+m_{s}\right)}-m_{s}\phi_{s}^{\prime }\rho_{f}^{\left(M_{f}+m_{s}\right)}\geq M_{s}\phi_{s}\rho_{s}^{\left(M_{s}\right)} \end{array}$$

where the left-hand side is the new revenue of the seeker with *F* serving its $m_{s}$ users and the right-hand side is the current revenue where the seeker serves all $M_{s}$ users. On the left-hand side, the first term is the revenue from serving $M_{s}-m_{s}$ users by the seeker itself (at rate $\rho_{s}^{\left(M_{s}-m_{s}\right)}$ bps per-user), the second term is the revenue from serving $m_{s}$ users by the feeder, and the third term is the maximum payment the seeker pays to the feeder calculated with the seeker’s reservation price.

From Equation (3), the upper-bound of the seeker’s reservation price is given as 

(4) $$\begin{array}{c} \phi_{s}^{\prime }\leq \frac{\phi_{s}\left(\left(M_{s}-m_{s}\right)\rho_{s}^{\left(M_{s}-m_{s}\right)}+m_{s}\rho_{f}^{\left(M_{f}+m_{s}\right)}-M_{s}\rho_{s}^{\left(M_{s}\right)}\right)}{m_{s}\rho_{f}^{\left(M_{f}+m_{s}\right)}} \end{array}$$

We take the seeker’s reservation price, $\phi_{s}^{\prime }$, to be the price where it is not improving its revenue, but only meeting the minimum rate requirement. Hence, $\phi_{s}^{\prime }$ is equal to the upper-bound given above.

The feeder *F* currently serves $M_{f}$ users at price $\phi_{f}$. It can serve all its $M_{f}$ users at the average per-user data rate higher than the minimum rate requirement, $\rho_{f,min}$. It can support additional $m_{s}^{*}$ users of *S* if $\rho_{f}^{\left(M_{f}+m_{s}^{*}\right)}\geq \rho_{f,min}$, “condition to be a feeder”. Hence, if $m_{s}\leq m_{s}^{*}$, then it can support an additional $m_{s}$ users, otherwise at most $m_{s}^{*}$ users.

In the best case, by serving additional $m_{s}$ users of *S*, *F* has lower average per-user data rate at $\rho_{f}^{\left(M_{f}+m_{s}\right)}$. Hence, it must be compensated by the seeker. The feeder also has its own reservation price, $\phi_{f}^{\prime }$, which is the minimum price that the feeder will accept from the seeker. The feeder has no need to improve its average per-user data rate since it already meets the minimum rate, i.e.,

(5) $$\begin{array}{c} \rho_{f}^{\left(M_{f}+m_{s}\right)}\geq \rho_{f,min} \end{array}$$

Its only motivation to collaborate with the seeker is in improving the revenue from utilizing available spectrum. That is, the feeder will agree to serve $m_{s}$ users of the seeker as long as its new revenue is better than the current revenue, i.e.,

(6) $$M_{f}\phi_{f}\rho_{f}^{\left(M_{f}+m_{s}\right)}+m_{s}\phi_{f}^{\prime }\rho_{f}^{\left(M_{f}+m_{s}\right)}\geq M_{f}\phi_{f}\rho_{f}^{\left(M_{f}\right)}$$

where the right-hand side is the current revenue of the feeder *F* while serving $M_{f}$ users at rate $\rho_{f}^{\left(M_{f}\right)}$. For the new revenue of the feeder *F* on the left-hand side, the first term is the revenue generated from serving $M_{f}$ users at the new lower rate $\rho_{f}^{\left(M_{f}+m_{s}\right)}$ and the second term is the minimum acceptable payment from the seeker at the feeder’s reservation price.

From Equation (6), the lower-bound of the feeder’s reservation price is given as

(7) $$\begin{array}{c} \phi_{f}^{\prime }\geq \frac{M_{f}\phi_{f}\left(\rho_{f}^{\left(M_{f}\right)}-\rho_{f}^{\left(M_{f}+m_{s}\right)}\right)}{m_{s}\rho_{f}^{\left(M_{f}+m_{s}\right)}} \end{array}$$

Note that the feeder’s reservation price, $\phi_{f}^{\prime }$, in Equation (7) is always greater than zero, since $\rho_{f}^{\left(M_{f}\right)}\geq \rho_{f}^{\left(M_{f}+m_{s}\right)}$. Similar to the seeker’s case, we take the feeder’s reservation price to be its lower-bound which is the price where the feeder is indifferent between serving the seeker’s $m_{s}$ users or not.

The agreement price, $\phi_{a}$, at which both the seeker and the feeder accept payment to have additional $m_{s}$ users of the seeker to be served by the feeder must be negotiated between them. However, for the negotiation to be successful, the necessary condition is that the seeker’s reservation price must be greater than the feeder’s reservation price and the agreement price is in between. That is,

(8) $$\begin{array}{c} \phi_{f}^{\prime }\leq \phi_{a}\leq \phi_{s}^{\prime } \end{array}$$

Here, the price range in Equation (8) signifies the zone where an agreement payment can be met which both negotiators, the seeker and the feeder, can agree to.

### 3.2. Negotiation for Collaboration

Since for simplicity we are considering only two MNOs, where one is a seeker and the other a feeder, we consider one-to-one negotiation, often termed as bilateral negotiation [33], which involves two participants (a seeker and a feeder) during a negotiation process (negotiation trade). In such negotiation, which is assumed to follow an alternating-offers protocol, the participating MNOs would exchange proposal offers that represents some acceptable payments until either an agreement is reached or the negotiation terminates with a failure.

Now, we develop a model in which the negotiators, a seeker and a feeder, can mutually influence each other in a negotiation trade. Let a negotiation trade round be denoted by c∈{1,…,C}, where *C* is the maximum number of rounds. At round *c*, negotiators, the seeker and the feeder, in the negotiation trade, alternatively propose and respond to an offer, $\phi_{sf}^{c}$ or $\phi_{fs}^{c}$, respectively. Following the negotiation strategy in [33,34], the offers at round *c* from the seeker and the feeder are assumed to follow:

(9) $$\phi_{sf}^{c}=\phi_{\min}+\alpha_{s}^{c}\left(\phi_{s}^{\prime }-\phi_{\min}\right)$$

(10) $$\phi_{fs}^{c}=\phi_{\max}-\alpha_{f}^{c}\left(\phi_{\max}-\phi_{f}^{\prime }\right)$$

where $\phi_{\min}$ and $\phi_{\max}$ are initial offers of the seeker and the feeder, respectively, and the pace of the negotiation trade is specified by the function $\alpha_{i}^{c},i\in \left\{s,f\right\}$. Since we are only interested in showing that the resource sharing is useful for both MNOs, we use a simple polynomial pacing function $\alpha_{i}^{c}={\left(\frac{c}{C}\right)}^{\frac{1}{\zeta_{i}}}$, where $\zeta_{i}>0$ determines the negotiation trade pace along time. With $0<\zeta_{i}<1$, a negotiator takes a Boulware strategy [35], i.e., tends to maintain an offered price until the time is almost exhausted, and then concedes to the reservation price quickly. With $\zeta_{i}>1$, a negotiator takes a Conceder strategy [36], i.e., goes to the reservation price rapidly and early. Despite the value of $\zeta_{i}$, with a constant reservation price, the offer from seeker (feeder) monotonically increases (decreases) as the negotiation rounds progresses.

Now, we model the response of the seeker and the feeder to an offer in a negotiation trade such that at round *c*,

- Response to an offer by the seeker MNO, $RO_{s}$:

(11) $$RO_{s}=\left\{\begin{array}{c} \mathrm{accept};\;\mathrm{if}\;\phi_{sf}^{c+1}\geq \phi_{fs}^{c}\\ \mathrm{reject and counter-offer};\;\mathrm{if}\;\phi_{sf}^{c+1}<\phi_{fs}^{c} \end{array}\right.$$

- Response to an offer by the feeder MNO, $RO_{f}$:

(12) $$RO_{f}=\left\{\begin{array}{c} \mathrm{accept};\;\mathrm{if}\;\phi_{fs}^{c+1}\leq \phi_{sf}^{c}\\ \mathrm{reject and counter-offer};\;\mathrm{if}\;\phi_{fs}^{c+1}>\phi_{sf}^{c} \end{array}\right.$$

Here, the negotiator’s response to an offer at each round follows an alternating-offers protocol that includes: *accept* and *reject and counter-offer*. From Equations (11) and (12), the negotiators propose and respond alternatively, until one accepts an offer or the negotiation trade reaches the maximum limit of *C* rounds.

## 4. Simulation Results

We evaluated the performance of collaboration among two MNOs, a seeker and a feeder. The simulations were performed in MATLAB version R2017b (9.3.0.713579). We considered deployment of small cells that belong to two MNOs in a cluster [37]. The small-cell cluster consists of small cells randomly deployed in a circular area of radius R=100 m. MSs were randomly distributed within circular coverage of radius r=30 m of small cells, i.e., within the coverage range of SBSs. The propagation model considered is 3GPP Model 1 (Table A.2.1.1.2-3) in [38], where the channel gains include path-loss, lognormal shadowing, and multipath Rayleigh fading. For simplicity, in our simulation, we considered channel bandwidth of B=1.4 MHz with corresponding number of subchannels K=6 and maximum transmit power of SBSs set to 20 dBm [39]. The numbers of deployed SBSs by seeker and feeder were set to 10 each. To evaluate the performance of the proposed mechanism, we considered the radio resources of the seeker and the feeder to be densely and sparsely populated, respectively. The numbers of MSs belonging to seeker and feeder, $\left(M_{s},M_{f}\right)$, were set to (100,10). The average per-user data rate threshold was set to 0.5 Mbps per-user. Assuming rational behavior of the participants in a negotiation trade, we considered the initial offers from seeker and feeder to be $\phi_{\min}=0$ and $\phi_{\max}=2$, respectively. As the negotiation trade between a seeker and a feeder is a time-constrained domain, we limited the maximum number of rounds to C=10 with the seeker initiating a proposal offer.

### 4.1. Effects of Strategy in Proposal Offers

**Figure 1:**
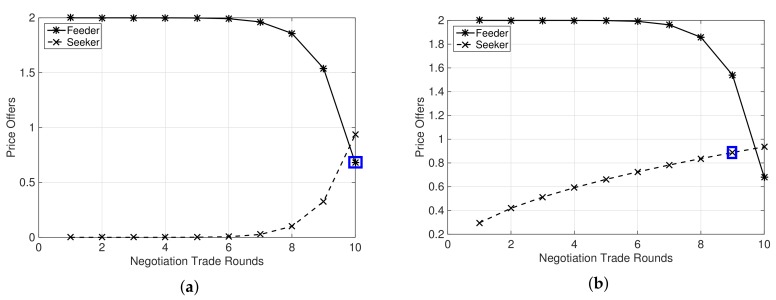

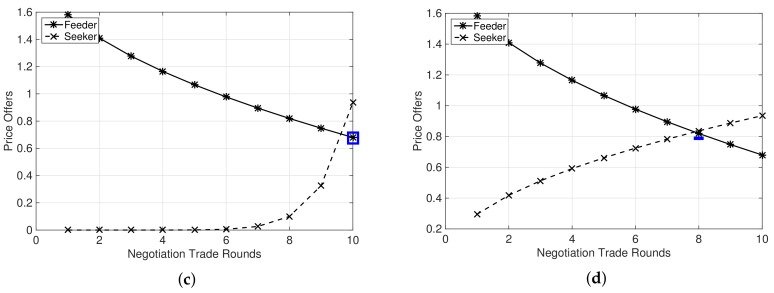
Proposal offers of a feeder and a seeker MNO depending upon values of $\zeta_{f}$ and $\zeta_{s}$, i.e., $0\;<\;\zeta_{i}\;<\;1$ and $\zeta_{i}>1$, and $M_{f}=10$ and $M_{s}=100$. A case of agreement in a negotiation trade, where agreement price follows $\phi_{f}^{\prime }\leq \phi_{a}\leq \phi_{s}^{\prime }$. (**a**) $\zeta_{f}=0.1$ and $\zeta_{s}=0.1$; (**b**) $\zeta_{f}=0.1$ and $\zeta_{s}=2$; (**c**) $\zeta_{f}=2$ and $\zeta_{s}=0.1$; (**d**) $\zeta_{f}=2$ and $\zeta_{s}=2$.

Figure 1a–d shows proposal offers made by feeder and seeker MNOs in the negotiation trade by following either Boulware strategy or Conceder strategy. The numbers of MSs belonging to the feeder and the seeker MNOs were fixed to 10 and 100 respectively. When both participants follow Boulware strategy, $\zeta_{f}=0.1$ and $\zeta_{s}=0.1$, we can notice that they maintain their offers until the negotiation trade round almost reaches the maximum limit *C* and then concede to their respective reservation prices when the negotiation trade reaches the limit. If any either of the participants follows a Conceder strategy, as shown in Figure 1b–d, the participant’s offer goes to their respective reservation prices quickly and much earlier in the negotiation trade of *C* rounds. In addition, the intersection point of the proposal offers happens as the number of feeder MSs, $M_{f}$, is comparatively less than the number of MSs that would provide them with improvement in revenue from their available network resources. Figure 1a–d signifies that an agreement payment is achievable in this negotiation trade of *C* rounds because a proposal offer of either feeder or seeker is favorable to the others following response strategy in Equations (11) and (12), and also noting the fact that the agreement price satisfies the necessary condition in Equation (8), i.e., $\phi_{f}^{\prime }\leq \phi_{a}\leq \phi_{s}^{\prime }$, for the negotiation to be successful.

**Figure 2:**
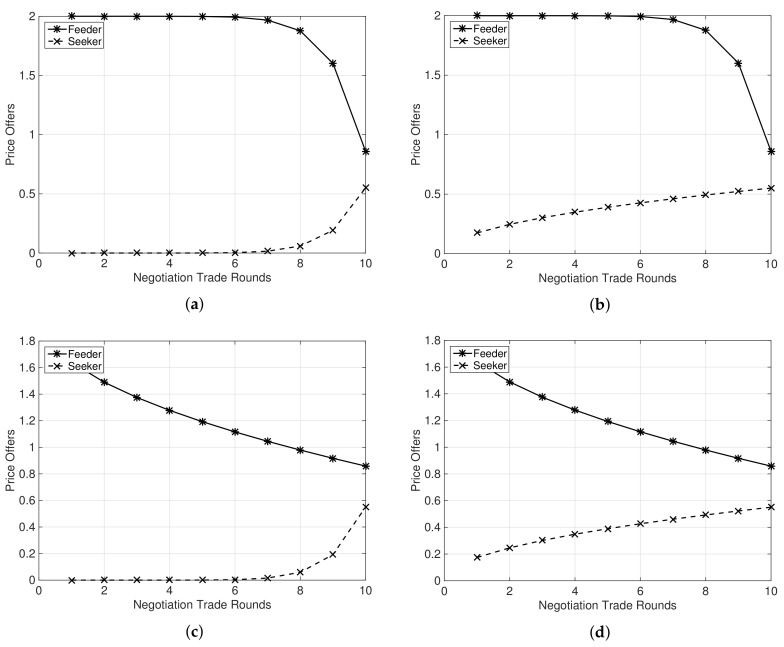
Proposal offers of a feeder and a seeker MNO depending upon values of $\zeta_{f}$ and $\zeta_{s}$, i.e., $0\;<\;\zeta_{i}\;<1$ and $\zeta_{i}>1$, and $M_{f}=50$ and $M_{s}=100$. A case of no agreement in a negotiation trade. (**a**) $\zeta_{f}=0.1$ and $\zeta_{s}=0.1$; (**b**) $\zeta_{f}=0.1$ and $\zeta_{s}=2$; (**c**) $\zeta_{f}=2$ and $\zeta_{s}=0.1$; (**d**) $\zeta_{f}=2$ and $\zeta_{s}=2$.

Figure 2a–d shows a case of no agreement in a negotiation trade. Here, we considered that the number of feeder MSs, $M_{f}$, is comparatively higher than the previous plots in Figure 1, i.e., $M_{f}=50$. In this case, although the feeder and the seeker MNOs enter a negotiation trade, no agreement can be reached upon any of the proposal offers. Notably, it does not satisfies the necessary condition for the negotiation to be successful in Equation (8), i.e., for the negotiation to be successful, the seeker’s reservation price needs to be higher than the feeder’s reservation price and the agreement price is in between, $\phi_{f}^{\prime }\leq \phi_{a}\leq \phi_{s}^{\prime }$.

### 4.2. Effects in Revenues

**Figure 3:**
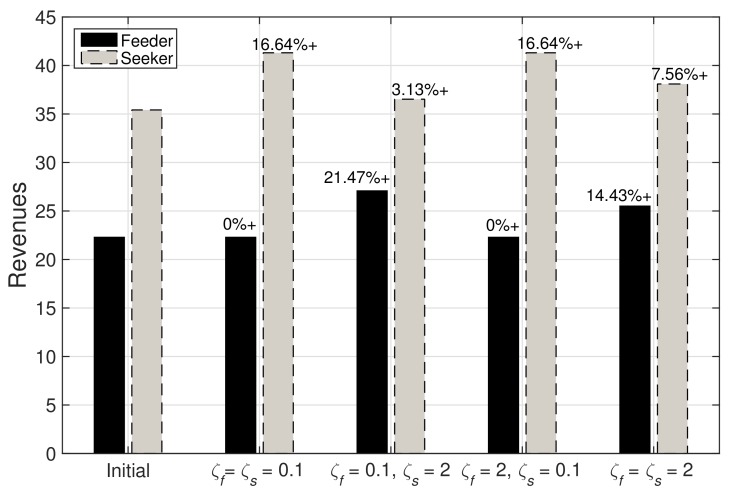
Revenues of the feeder and the seeker MNOs depending upon values of $\zeta_{f}$ and $\zeta_{s}$, i.e., $0\;<\;\zeta_{i}\;<1$ and $\zeta_{i}>1$, and $M_{f}=10$ and $M_{s}=100$.

Figure 3 plots the revenues obtained by the feeder and the seeker MNOs after successful agreement on a proposal offer in a negotiation trade. Once a proposal offer from either a feeder or a seeker is accepted in the negotiation trade, as shown in Figure 1, and a collaboration is formed, the plots show that both participants would at least benefit with an increment in revenues from their initial revenues. However, the rates of increment from the initial revenues are dependent on strategies: $\zeta_{f}$ and $\zeta_{S}$ values. Notice that, if either of the participant is taking Boulware strategy, then negotiation agreement could be met at much better offers as compared to its reservation price, and this participant could generate a much better revenue. However, it also depends on the strategy taken by the other participant. In addition, the revenues in Figure 1 for $\zeta_{f}=\zeta_{s}=0.1$ and $\zeta_{f}=2,\;\zeta_{s}=0.1$ are similar. This is because the agreement price $\phi_{a}$ for both cases are the same, i.e., the reservation price of the feeder, as seen in Figure 1a,c. A more general scenario would be that the seeker chooses Conceder strategy, goes to the reservation price rapidly and early, and the feeder chooses Boulware strategy, maintains an offered price until the end of the negotiation round, as shown in Figure 1b. In such scenario, we can notice from the plot in Figure 3 that the feeder has the most increment in their revenue while the seeker also obtains some increment. Hence, both the MNOs will have a *win–win* policy for collaboration formation at the agreement price.

### 4.3. Effects in Average Per-User Data Rate

**Figure 4:**
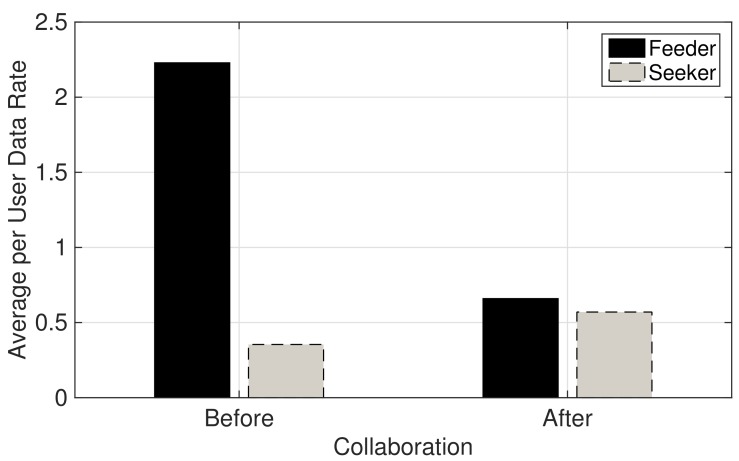
Before and after collaboration average per-user data rate of the feeder and the seeker MNOs.

Figure 4 plots before and after collaboration average per-user data rate obtained by the users of the feeder and the seeker MNOs. As shown in the figure, the average per-user data rate of the feeder MNO after collaboration is decreased while the seeker MNO’s average per-user data rate is increased above the threshold value of 0.5 Mbps per-user. This is because both MNOs try to serve possible maximum number of users to increase their revenues while maintaining the average per-user data rate threshold. Following this, the feeder MNO serves additional MSs of the seeker MNO using its own network resources. The seeker MNO improves the average per-user data rate by reducing its number of MSs to serve by transferring some of its MSs to get service by utilizing the network resources of the feeder MNO.

### 4.4. Effects in System Capacity

**Figure 5:**
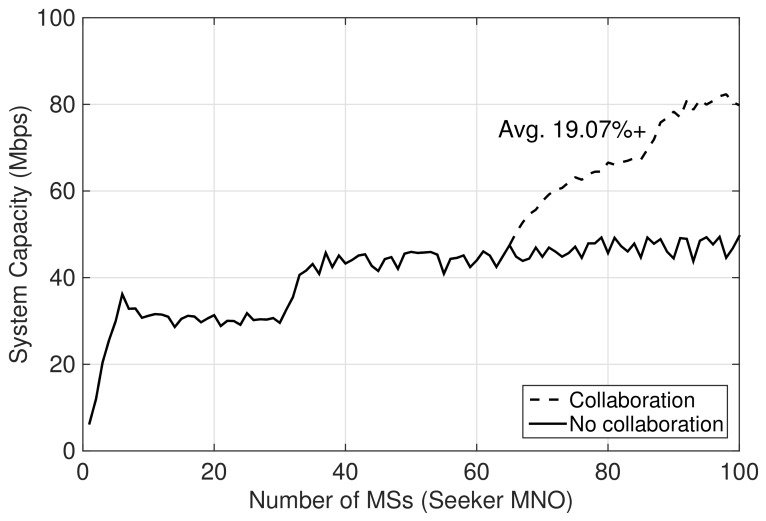
Before and after collaboration comparison of system capacity of the seeker MNO.

Figure 5 shows the system capacity of the seeker MNO that is obtained after collaboration and compared with the case of no collaboration. In the figure, the collaboration improves the system capacity of the seeker MNO by enabling some of its MSs to be served by utilizing the feeder MNO’s network resources. However, the number of MSs being served by the feeder could be limited depending on the number of MSs belonging to the feeder MNO, $M_{f}$. In this regard, the system capacity of the seeker MNO would be dependent on $M_{f}$ since it dictates how many extra MSs the feeder would be able to serve using its network resources while maintaining the average data rate per-user. From simulation, the average number of MSs that the feeder could serve while maintaining the average data rate per-user is 56. Hence, for given $M_{f}=10$, the feeder could collaborate to serve extra MSs belonging to the seeker MNO, i.e., at most $m_{s}^{*}=46$. The seeker MNO requires at most $m_{s}=35$ of its users to be served by the feeder MNO to maintain its average data rate per-user. Since $m_{s}\leq m_{s}^{*}$, the feeder MNO can serve all additional 35 MSs belonging to the seeker MNO. In Figure 5, the system capacity of seeker MNO after collaboration is improved as compared to no collaboration on average by 19.07%.

**Figure 6:**
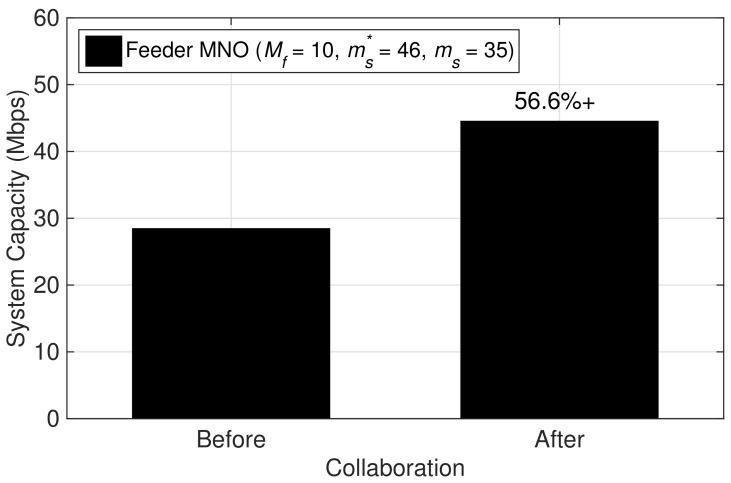
Before and after collaboration comparison of system capacity of the feeder MNO.

Similarly, for the feeder MNO, the network resources are well utilized after serving additional MSs belonging to the seeker MNO, i.e., $m_{s}=35$. Comparing the results in Figure 6, the system capacity of the feeder MNO after collaboration is improved as compared to before collaboration by 56.6%, and hence avoids under-utilization of network resources. Here, the total number of users being served from the feeder MNO’s network resources is increased from $M_{f}=10$ to $M_{f}+m_{s}=45$. Moreover, the feeder MNO experience lower average per-user data rate at $\rho_{f}^{\left(M_{f}+m_{s}\right)}$ while maintaining the average per-user data rate than the minimum rate requirement, $\rho_{f,min}=0.5$ Mbps, as shown in Figure 4. In addition, the revenues obtained by the feeder MNO after collaboration is not less than the initial revenues, as shown in Figure 3.

### 4.5. Comparison with Shared Networks

**Figure 7:**
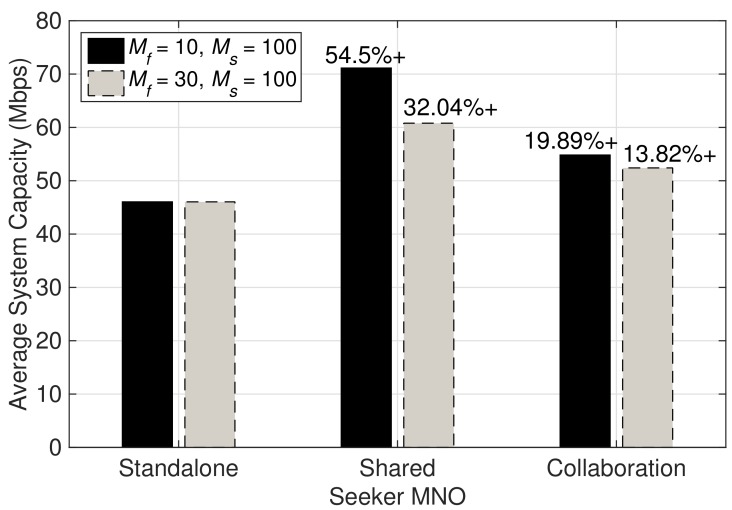
Comparison of average system capacities of the seeker MNO while standalone, shared, and in collaboration with the feeder MNO.

**Figure 8:**
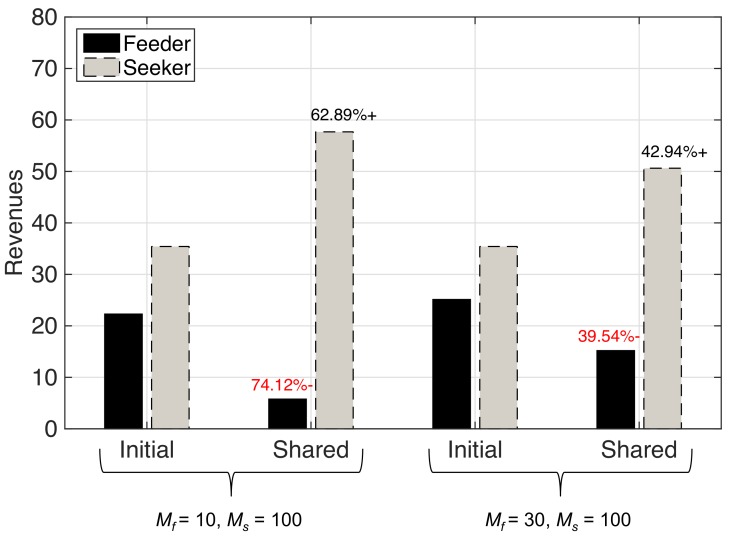
Comparison of initial revenues with revenues obtained from sharing network resources of the feeder and the seeker MNOs.

Figure 7 compares the average system capacity of the seeker MNO that is obtained while in standalone mode, sharing the network resources with the feeder MNO, and in collaboration with the feeder MNO, for the two cases: (i) $M_{f}=10$; and (ii) $M_{f}=30$, given $M_{s}=100$ remains fixed. Significant improvement in average system capacity can be observed for both cases while sharing the network resources. However, note that there is a significant drop in the average system capacity by 22.46% when $M_{f}$ is increased from 10 to 30. Sharing the network resources provide with improved average system capacity for both cases as compared to collaboration. However, this improvement comes from the fact that the whole network resources of the feeder MNO is utilized by the seeker MNO. Since the number of MSs belonging to the seeker MNO is comparatively large compared to that of the feeder MNO, the revenues obtained by them following Equation (1) would not be favorable to either of them. The indifference in the revenues obtained by the feeder and seeker MNO for the two cases can be observed in Figure 8. Unsurprisingly, there is a large decline in revenues of the feeder MNO while sharing the network resources with the seeker MNO in both cases. Obviously, such a result would not motivate the feeder MNO to share its network resources with the seeker MNO, and hence signifies the importance of considering negotiation to reach an agreement with suitable MNO that could offer each party collaboration gains as a motivation behind sharing network resources. In addition, recall from Figure 3 that the revenues obtained by the feeder and the seeker MNO after collaboration for the case of $M_{f}=10$ and $M_{s}=100$ are not less than their initial revenues.

### 4.6. Comparison with Different Resource Allocation Schemes

As shown in Figure 7, we observed that the small number of $M_{f}$ would result in significant improvement in the system capacity for the seeker MNO while collaborating as compared to no collaboration, since more MSs belonging to the seeker could be served by the feeder’s network resources. However, an increment in average system capacity would also depend upon the schemes used for resource allocations. To compare and evaluate the performance of multi-operator small cells collaboration with different resource allocation schemes, we considered the best-response (BR) strategy based different schemes, namely BR (Simultaneous), BR (Sequential), and marginal contribution-based best-response (MCBR) with neighborhood and complete information [40,41]. We averaged the performance for 200 randomly generated deployments of small cells that belong to the two MNOs in a cluster and its associated MSs.

**Figure 9:**
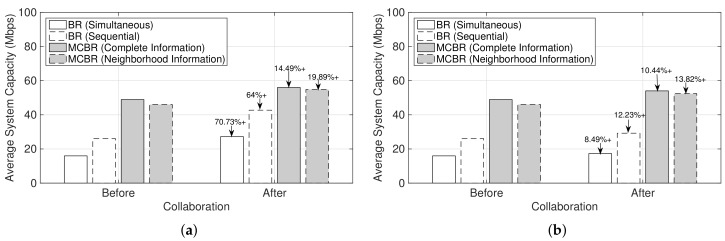
Average system capacities comparison of the seeker MNO before and after collaboration. (**a**) $M_{f}=10$ and $M_{s}=100$; (**b**) $M_{f}=30$ and $M_{s}=100$.

In Figure 9a,b, all the schemes improve average system capacity of the seeker MNO with collaboration as compared to no collaboration. Moreover, as the number of $M_{f}$ increases (compare Figure 9a,b), the corresponding average system capacity improvement after collaboration is decreased for all schemes. This result is consistent with the observation in Figure 7 and due to the fact that the feeder MNO could serve fewer MSs belonging to the seeker. Figure 9a,b also signifies the importance of resource allocation schemes. As compared to the BR strategy schemes, both MCBR schemes provide significant improvement in average system capacity for both before and after collaboration. Thus, the increment of $M_{f}$ from 10 to 30 does not have significant effect on the average system capacity of a seeker MNO while using MCBR schemes.

## 5. Conclusions

In this paper, a mechanism for multi-operator small cells collaboration for network resource management among multiple MNOs is presented. Depending upon policy profile of MNOs, a negotiation trade can be initiated between a seeker and a feeder MNO. An agreement upon a proposal offer indicates that both participants in the negotiation trade could benefit while collaborating: the seeker would benefit by enabling some of their subscribers to get served by network resources of the feeder MNO, thus, improving average per-user data rate of their users, while the feeder MNO generates more revenues from proper utilization of their network resources by serving additional users. Simulation results show that collaborative resource management is able to achieve significant performance gain as compared to sharing the network resources for several performance metrics. Multi-operator small cells collaboration formulates mutual agreement among MNOs prior to the usage of network resources, and is able to enhance services to users with an improved network resources availability, improve MNOs’ revenue generated from an increased market share, avoid under-utilization of network resources, and create an opportunity for ubiquitous access to wireless coverage and connectivity by maintaining SCNs service to subscribers.

## Abbreviations

The following abbreviations are used in this paper:

3GPP	3rd Generation Partnership Project
BPS	Bits per Second
BR	Best Response
BS	Base Station
BYOD	Bring-Your-Own-Device
CMM	Collaboration Management Module
C-RAN	Cloud-Radio Access Network
GWCN	Gateway Core Network
MBS	Macrocell Base Station
MCBR	Marginal Contribution-Based Best Response
MNO	Mobile Network Operator
MOCN	Multi-Operator Core Network
MS	Mobile Station
RAN	Radio Access Network
SBS	Small-cell Base Station
SCN	Small Cell Network
